# Recent progress in the treatment for unresectable biliary tract cancer

**DOI:** 10.1002/ags3.12710

**Published:** 2023-06-29

**Authors:** Itaru Endo

**Affiliations:** ^1^ Department of Gastroenterological Surgery Yokohama City University Graduate School of Medicine Yokohama Japan

## Abstract

Recent changes in the treatment of unresectable BTC have been remarkable. More recently, triple combination therapy with GC and S‐1 (GCS) and GC and nab‐Paclitaxel was used. Also, immune checkpoint inhibitors have been increasingly used for BTC.

Approximately 22 000 cases of gallbladder and extrahepatic cholangiocarcinoma are diagnosed every year in Japan,^1^ which ranks 13th among all carcinomas and has shown an increasing trend in recent years. At 17 773 (2020), the number of deaths is sixth, and has plateaued over the past few years. The 5‐year relative survival rate is 24.5%, which is the second worst after pancreatic cancer. Surgical resection is considered the only potentially curative treatment for biliary tract cancer (BTC), but most patients with BTC (60%–70%) have advanced cancer that is not amenable to surgical resection because of distant metastases or local progression at initial presentation. Patients with metastases are considered to have a particularly poor prognosis, as the median survival in such cases is less than 8 months.^2^ Efforts to increase the number of long‐term survivors of BTC have been directed toward the establishment of safe surgical techniques, postoperative adjuvant chemotherapy, and resection after preoperative treatment for borderline unresectable/unresectable patients.^3^, ^4^, ^5^ Ioka et al.^6^ wrote an elaborate review of perioperative chemotherapy and discussed future prospects, which were very informative.

Recent changes in the treatment of unresectable BTC have been remarkable. Primary treatment of unresectable BTC started with fluorouracil or gemcitabine (GEM) alone. Later, GEM plus cisplatin (GC) therapy was established as the current standard of care, but achieved only a slight median overall survival prolongation (11.7 vs. 8.1 months) compared with GEM monotherapy.^7^ Subsequently, the FUGA‐BT trial was conducted in Japan, comparing GC therapy with the combination of GEM and S‐1 (an oral fluoropyrimidine combination consisting of tegafur, gimeracil, and oteracil) (GS). The results of that study revealed that GS therapy was non‐inferior to GC therapy and well tolerated.^8^ More recently, triple combination therapy with GC and S‐1 (GCS) was used as first‐line therapy for advanced BTC in Japan.^9^ The superiority of GCS over GC therapy was demonstrated in the phase III KHBO1401‐MITSUBA study, and good antitumor efficacy was also seen (Table 1). On the other hand, GCA therapy (GC + nabpaclitaxel (Abraxane)) was used in the US, demonstrating excellent median overall survival of 19.2 months (Table 1).^10^ Unfortunately, the results of a randomized phase III trial comparing GCA and GC therapy presented at the 2023 American Society of Clinical Oncology Gastrointestinal Symposium failed to show a significant difference between the two groups. However, in a subset analysis, GCA versus GC was reported to be 19.2 versus 13.7 months for locally advanced cancer and 17.0 versus 9.3 months for gallbladder cancer, showing some promising aspects.^11^ The authors stated that the results of sub‐genomic analyses are also expected in the future. Therefore, GCA therapy could become a neoadjuvant therapy for “borderline resectable” high‐risk patients.

**TABLE 1:** Recent changes in the anti‐tumor drug treatment of unresectable biliary tract cancer.

Author	Year	Regimen	Number of patients	% Asian ethnicity	ICC (%)	ECC (%)	GBC (%)	Metastatic (%)	Locally advanced (%)	Median PFS	CR (%)	PR (%)	ORR	DCR	mOS	2 year OS rates (%)	Grade 3 or higher AE
Valle	2010	GC	204	NA	59.8		35.8	73	27	8				81.4	11.7	≒ 15	70.7
		G	206	NA	57.8		36.9	76.2	23.8	5				71.8	8.1	≒ 5	68.8
Morizane	2019	GS	179	100	25	33	39	61	18	6.8			29.8		15.1	≒ 25	29.9
		GC	175	100	29	28	39	61	18	5.8			32.4		13.4	≒ 25	35.1
Shroff	2019	GCA	60	NA	63	15	22	78	22	11.8	0	45	45	84	19.2	≒ 38	58
Ueno	2019	GC + Nivolumab	30	100						4.2			36.7		15.4		77
Oh	2022	GC + Durvalmab	341	52.2	55.7	19.4	24.9	88.9	11.1	7.2	2.1	24.6	26.7	85.3	12.8	24.9	75.7
		GC	344	57	56.1	18.9	25	83.1	16.6	5.7	0.6	18.1	18.7	82.6	11.5	10.4	77.8
Shi	2023	GEMOX + Toripalimab + Lenvatinib	30	100	100					10.2		76.7	80	93.3	22.5	49.8	56.7
Kelly	2023	GC + Pembrolizumab	533	46	60	18	22	89	11	6.5	2	27	29	75	12.7	25	79
		GC	536	47	58	20	22	88	12	5.6	1	27	28	75	10.9	18	75
Ioka	2023	GCS	123	100	35	30.9	34.1	59.3	14.6	7.4	3	38	41	79	13.5	≒ 25	
		GC	123	100	28.5	35	32.5	49.6	26	5.5	1	14	15	62	12.6	≒ 20	
Shroff	2023	GCA	294							8.2			34		14		60
		GC	147							6.4			25		12.7		45
El‐Khoueiry	2023	GC + Atezolizumab + Bevacizumab	79	43	54	19	27	82	18	8.4			24		NR		73
		GC + Atezolizumab	83							7.9			25		11.4		74

Abbreviations: A, Abraxane (nab‐paclitaxel); AE, adverse event; C, cisplatin; CR, complete response; DCR, disease control rate; ECC, extrahepatic cholangiocarcinoma; G, gemcitabine; GBC, gallbladder cancer; ICC, intrahepatic cholangiocarcinoma; mOS, median overall survival; NA, not available; ORR, objective response rate; OX, oxaliplatin; PFS, progression free‐survival; PR, partial response; S, S‐1.

Recently, immune checkpoint inhibitors (ICIs) have been increasingly used for BTC.^12^ Unfortunately, as reported by Ueno et al.,^13^ GC + nivolumab did not provide the expected results. However, two phase III trials, KEYNOTE‐966 and TOPAZ‐1, met their primary end points, thereby establishing GC + ICI as first‐line therapy for advanced BTC.^14^, ^15^ However, there were a few points of concern in the subgroup analysis: GC + durvalumab (GCD) was more effective in Asians and GC + pembrolizumab (GCP) was more effective in non‐Asians. Careful interpretation is needed because different trials had different backgrounds. In addition, BTC often requires biliary drainage. Cholangitis frequently occurs in such instances and requires antimicrobial administration; the efficacy of ICIs is known to be attenuated by the administration of antimicrobials.^16^ Furthermore, regimens such as gemcitabine+oxaliplatin (GEMOX) + toripalimab + lenvatinib,^17^ and GC+atezolizumab+bevacizumab^18^ are under development. What will be the future of first‐line treatment of BTC in Japan? Over the next few years, we will acquire more experience with the various first‐line agents mentioned above, after which, we will be able to make a decision.

BCT has become the mainstream of modern oncological care, but there is still much work to be done, such as comprehensive genome panel (CGP) profiling after disease recurrence, which could lead to the discovery of effective molecularly targeted therapies if promising gene mutations are found. Patients with BTC have a relatively high chance of finding druggable mutations by CGP profiling. In addition to standard treatment, molecular profiling and expert opinions at large academic centers are needed to ensure that patients receive the best possible treatment. We will keep an eye on the progress of BTC treatment for some time to come.

## FUNDING INFORMATION

This editorial did not receive any specific grant from funding agencies in the public, commercial, or nonprofit organizations.

## CONFLICT OF INTEREST STATEMENT

Author I.E. (Itaru Endo) was supported by scholarship donations from Taiho Pharmaceutical, Ono Pharmaceutical, MSD Pharmaceutical, Chugai Pharmaceutical, and Eisai Co Ltd., and is currently an editorial board member of *AGS*. I.E. was not involved in the review of this manuscript or in the selection of reviewers.

## ETHICS STATEMENTS

Approval of the research protocol: N/A.

Informed consent: N/A.

Registry and the registration no. of the study/trial: N/A.

Animal studies: N/A.
